# Comment on “Estimating serum‐ionized magnesium concentration in hemodialysis patients”

**DOI:** 10.1111/hdi.12975

**Published:** 2021-07-27

**Authors:** Dennis G. Begos, Anne Deutsch

**Affiliations:** ^1^ Department of Medical and Scientific Affairs, Nova Biomedical Waltham Massachusetts USA; ^2^ German Society for Magnesium Research/Deutsche Gesellschaft für Magnesiumforschung Tutzing Germany

To the Editors:

We read with interest the recent paper in your journal by Holzmann‐Littig and colleagues entitled “Estimating serum‐ionized magnesium concentration in hemodialysis patients”.^1^ The authors concisely and expertly review the importance of measuring ionized magnesium (iMg) in hemodialysis patients, as iMg levels are well known to affect morbidity and mortality in this patient population.^2^, ^3^ The importance of measuring iMg is accurately highlighted in the paper, as iMg is the physiologically active component of serum Mg.^4^ The authors use the direct measurement of iMg using an ion‐sensitive electrode as the gold standard reference for iMg concentration to compare with their formula for calculating iMg.

In the introduction, the authors state that “…accurate measurement of Mg_ion_ is methodologically challenging and cost‐intensive in clinical practice.”.^1^ We believe that this statement is inaccurate and misleading for several reasons. The authors used a Nova Biomedical device, a Nova CRT 8 Electrolyte Analyzer as their reference analyzer for iMg. While this device uses an ion‐sensitive electrode and has been shown to be very accurate and precise, it was designed and manufactured over 40 years ago. Although it is a testament to the device that it still functions well, considerable advances in technology have been made in the intervening decades. Today, there are two devices for measuring iMg. One measures iMg along with four other electrolytes in 1 min. The other measures iMg, along with 22 other metabolites, blood gases, and CO‐oximetry. Both use a credit‐card sized cartridge to house all sensors. They are both cost‐effective and not a cost‐intensive solution for measuring iMg and are far more accurate and precise than estimating iMg using a formula.

The authors are to be commended for the work involved in developing and validating this formula. The authors state that “An equation containing three variables performed well both in terms of accuracy to estimate the ionized value and to predict normomagnesemia.” However, this formula has only been found to be 84% accurate in the external validation cohort, with an area under the curve (AUC) of only 0.78 for determining normomagnesemia.^1^ Additionally, it has only been studied to predict normomagnesemia, not hypo‐ or hypermagnesemia, limiting its usefulness in important clinical situations. In our estimation, this falls below an acceptable standard for clinical decision‐making and begs the question: “why not just measure ionized magnesium?”

## CONFLICT OF INTEREST

Both authors are employees of Nova Biomedical.
